# Golimumab in juvenile idiopathic arthritis-associated uveitis unresponsive to Adalimumab

**DOI:** 10.1186/s12969-021-00630-1

**Published:** 2021-08-21

**Authors:** Sofia Lanz, Gerald Seidel, Andrea Skrabl-Baumgartner

**Affiliations:** 1 Division of General Pediatrics, Department of Pediatrics and Adolescent Medicine, Medical University Graz, Graz, Austria; 2 Department of Ophthalmology, Medical University Graz, Graz, Austria

**Keywords:** Juvenile idiopathic arthritis, Refractory uveitis, Golimumab, Adalimumab, Treatment failure

## Abstract

**Objective:**

To assess the efficacy of golimumab (GLM) as a treatment option for juvenile idiopathic arthritis (JIA)-associated uveitis refractory to adalimumab (ADA).

**Methods:**

Retrospective single-centre study including patients with JIA receiving GLM for active uveitis after failing ADA. JIA- and uveitis-related data, including intraocular inflammation, best-corrected visual acuity, corticosteroid-sparing potential, and ocular complications were evaluated at start of GLM treatment, at 1 month and 3 months, and every 3 months thereafter during GLM administration. We further investigated the association of response to GLM with primary and secondary failure of ADA treatment.

**Results:**

Ten patients were studied, all female (17 affected eyes, mean age 14.3 + 6.7 yrs., mean follow-up 25.2 + 21.7 mos). Two patients were switched to GLM because of primary non-response to ADA. Eight were switched because of loss of response (LOR).

In 5 of the latter LOR was associated with neutralizing anti-ADA-antibodies. Response to GLM was observed in all 8 patients with LOR, while the 2 patients with primary non-response to ADA also did not respond to GLM. Three of the 8 responders experienced LOR. At the end of follow-up 4 of the 5 remaining responders had achieved complete response. One had achieved partial response.

**Conclusion:**

GLM is an efficacious therapeutic option in patients who experience LOR to ADA. Our data indicate that patients without primary response to ADA should be rather switched to a biologic agent with a different mode of action instead of further blocking the TNF-alpha pathway.

## Background

Uveitis is a potentially blinding complication of juvenile idiopathic arthritis (JIA) [1–3]. Early and complete control of ocular inflammation in JIA is important. Treatment moves step-by step, starting with topical and/or systemic steroids and followed by conventional disease-modifying anti-rheumatic drugs (cDMARDs), usually methotrexate (MTX). In refractory cases treatment with anti-tumor necrosis factor (TNF)-α agents is recommended [4, 5]. Among these adalimumab (ADA), a fully humanised antibody against TNF-α and the only TNF-α inhibitor approved for uveitis as of 2021, has become the drug of choice. ADA shows efficacy in around 75% of patients in the first 18 months of treatment [6, 7]. In case of no response or loss of response to ADA, a switch to another biological agent is recommended [5, 8, 9]. However, the choice of biological drug *i*s not clearly defined. The question remains: Is persistence in an attack on the TNF- α pathway promising in these patients? In this regard data from small numbers of patients provide evidence that switching to a second anti-TNF agent may be beneficial [8, 10]. As such golimumab (GLM), another fully humanised anti-TNF-α monoclonal antibody approved for the treatment of polyarticular JIA, has shown promising results in small heterogeneous case series [11–13].

The aim of our study was to assess the effectiveness of GLM in patients with JIA-associated uveitis who had failed to respond to ADA. We further determined if patients with a primary non-response to ADA fared differently than those with a loss of response (LOR) to ADA.

## Patients and methods

We conducted a single-centre, retrospective study in patients with JIA-associated uveitis who were treated with GLM for active uveitis that had proved refractory to at least one cDMARD and to ADA. All patients were evaluated at the outpatient clinics of the uveitis unit and of the pediatric-rheumatology service at the Medical University of Graz / Austria. JIA was diagnosed according to the International League of Associations for Rheumatology classification [14]. Uveitis was defined and anatomically classified according to the recommendations of the Standardization of Uveitis Nomenclature (SUN) Working Group [15]. Clinical and laboratory data were evaluated at the start of GLM treatment, at 1 month and at 3 months, and every 3 months thereafter during GLM administration. All patients were recruited between March 2010 and May 2018. Patients were followed until April 2021.

Before the study began the institutional review board of the Medical University of Graz approved all study procedures, the study protocol, and the informed-consent form for off-label treatment with GLM. All patients and/or parents gave written informed consent.

### Uveitis outcome measurement

The main outcome measures were assessed at predefined time points as mentioned above and included intraocular inflammation as determined by anterior chamber cell count, best-corrected visual acuity (BCVA), corticosteroid-sparing potential, and ocular complications. Results of slit-lamp examination, applanation tonometry, ophthalmoscopy, fluorescein angiography, and spectral-domain optical coherence tomography were evaluated. Anterior chamber (AC) cells and vitreous haze (VH) were graded by SUN criteria [16]. BCVA was determined using a Snellen chart.

### Response to GLM treatment

Response was classified as complete, partial, or none (“non-response”, NR) at each time point. Complete response (CR) constituted achieving inactive uveitis, defined as 0+ cells in the AC (grade 0). Partial response (PR) was diagnosed in patients with improved uveitis, defined as a decrease in the level of inflammation, without achieving AC grade 0 status. Primary NR was diagnosed in patients without change in SUN score and an entry grade of 3 or higher or in patients with worsening activity, defined as either a two-grade increase in inflammation or an increase in inflammation to grade 4. Relapse of uveitis was defined as active inflammation after at least 3 months of inactivity [16]. With bilateral disease, the eye with the higher grade of uveitis at the start of GLM was assessed. Loss of response (LOR) was defined as secondary NR in patients who had initially responded to treatment but relapsed and who failed to improve under continued treatment with GLM despite intermittent intensifying concomitant therapy, such as local or systemic corticosteroids, followed by discontinuation of GLM. Corticosteroid-sparing potential was defined as the reduction of systemic and topical corticosteroid dose. Topical and systemic corticosteroid doses were recorded for all visits.

### Failure of ADA treatment

Primary non-response and loss of response to ADA were defined in the same way as for GLM. In those cases available, ADA trough levels and anti-ADA antibodies were measured by enzyme-linked immunosorbent assay. Values greater than 10 U/mL were considered as demonstrating anti-drug antibodies.

### Side effects of GLM treatment

During the study, the patients themselves and their parents were regularly asked to describe any unusual experiences or phenomena encountered. Their reports and descriptions were used to assess side effects.

### Statistical analysis

Testing for normal distribution was performed by Kolmogorov-Smirnov test and results were reported as mean + SD or as median (range) as appropriate. Visual acuity was compared with paired t-test. Statistical significance was defined as *p* < 0.05. A Kaplan-Meier plot was used to visualize time to treatment failure. All statistical analyses were performed using GraphPad Prism 9.1 (GraphPad, San Diego, CA).

## Results

### Patient characteristics at baseline

Ten patients (all female) were studied. The mean age was 14.3 + 6.7 years (range 3.25–21.92).

Mean age at onset of JIA was 2.96 + 1.26 years (range 0.75–5). JIA subtype was oligoarthritis in 9 patients and enthesitis-related arthritis in 1. Mean age at onset of uveitis was 5.24 + 2.53 year (range 2.25–9.92). Seven patients suffered from anterior uveitis and 3 from panuveitis. The disease was bilateral in 7 patients and unilateral in 3. The median time between onset of uveitis and start of GLM treatment was 8.95 + 5.96 years (range 1–17.42).

Before treatment with GLM, all patients had received at least one cDMARD, including MTX (*n* = 10), azathioprine (*n* = 4), mycophenolate mofetil (*n* = 3), sulfasalazine (*n* = 1), tacrolimus (*n* = 1), interferon-α (*n* = 1), and ADA. Before receiving ADA 4 patients had been treated with a biological DMARD other than ADA, including infliximab (*n* = 4) and etanercept (*n* = 1).

Failure of ADA treatment was classified as primary NR in 2 patients (20%) and as LOR in 8 (80%). Five patients had achieved complete response before experiencing LOR. Anti-ADA antibodies were associated with LOR in 5 of the 7 patients (71.4%) in whom these biomarkers were assessed (Table 1).

**Table 1:** Uveitis response to adalimumab and golimumab

Pat.	Duration of uveitis before GLM (yrs)	Previous therapy	Response to ADA	Con comitant cDMARD	Anti-drug Ab	Response to GLM	Con comitant cDMARD	Anti-drug Ab	Current biologic therapy, response
1	6.2	MTX, ADA	CR, LOR	–	pos	CR	–	neg	GLM
2	4.1	MTX, AZA, ADA	PR, LOR	AZA	neg	PR, LOR	AZA	neg	TFC, PR
3	12.7	MTX, AZA, ETA, SSZ, IFX, MMF, TCR, IFNα, ADA	PR, LOR	–	n.d.	PR	–	n.d.	GLM
4	1.0	MTX, ADA	CR, LOR	MTX	pos	CR	MTX	neg	GLM
5	10.9	MTX, ADA	CR, LOR	–	pos	CR	–	n.d.	GLM
6	17.4	MTX, MMF, ADA	CR, LOR	–	n.d.	CR	–	n.d.	GLM
7	12.3	MTX, ADA	CR, LOR	–	pos	PR, LOR	MTX	n.d.	ADA, CR
8	4.8	MTX, IFX, ADA	PNR	MTX	pos	PNR	MTX	n.d.	TCZ, CR
9	17.3	MTX, AZA, IFX, MMF, ADA	PR, LOR	AZA	n.d.	PR, LOR	MMF	n.d.	ABA, PR
10	2.9	MTX, AZA, IFX, ADA	PNR	MTX	neg	PNR	MTX	n.d.	TCZ, CR

*MTX* methotrexate, *AZA* azathioprine, *SSZ* sulfasalazine, *MMF* mycophenolatmofetil, *TCR* tacrolimus, *IFNα* interferon α, *ETA* etanercept, *IFX* infliximab, *ADA* adalimumab, *ABA* abatacept, *TCZ* tocilizumab, *TFC* tofacitinib, *anti-drug-Ab* anti-drug-antibodies, *CR* complete response, *PR* partial response, *PNR* primary non-response, *LOR* loss of response, *n.d.* not done

Routine drug monitoring in all ADA-treated patients was established in June 2011. Data for patients before that time are lacking.

Ocular complications at the start of GLM therapy were present in 8 patients. They included macular edema (*n* = 2), cataract (*n* = 4), glaucoma (*n* = 2), synechiae (*n* = 7), and band keratopathy (*n* = 2).

At baseline, an AC cell grade of 1+ was found in 4 patients, with grades of 2+ in 2 patients, 3+ in 2 patients, and 4+ in 2 patients.

### Treatment at baseline

Patients were treated with GLM in the standard dose of 50 mg subcutaneously every 4 weeks in patients weighing ≥40 kg and 30 mg/m2 body surface area in patients weighing ≤40 kg.

At the start of GLM treatment 6 of 10 patients (60%) were receiving concomitant immunosuppressive therapy with MTX (*n* = 4) or azathioprine (*n* = 2) at conventional doses. Table 1 shows any previous and concomitant immunosuppression for all patients.

Systemic corticosteroids were used in 5 patients (50%; median dose 0.38 mg/kg, range 0.23–0.52) and topical corticosteroids (prednisolone acetate 1%) in 9 patients (90%; median 3 drops/day, range 1–10).

### Response to GLM treatment

Median follow-up with GLM treatment was 25.2 months (range 6–66). Response was achieved in 6 of 10 patients (60%; CR *n* = 2, PR *n* = 4) at 1 month, in 8 of 10 patients (80%; CR *n* = 4, PR *n* = 4) at 3 months, in 7 of 10 patients (70%; CR *n* = 3, PR *n* = 4) at 6 months, in 6 of 8 patients (75%; CR *n* = 5, PR *n* = 1) at 9 months, in 5 of 6 patients (83%; CR *n* = 4, PR *n* = 1) at 12 months, and in 5 of 6 patients (83%; CR *n* = 5) at 18 months. A complete response persisted in all 5 at 24 months and 30 months.

Two patients were treated for longer than 60 months. At their final visit one of these patients continued in CR and the other, after experiencing a flare at 60 months, had responded again to GLM on assessment at 66 months. During the aggregated 248 treatment months 19 flares occurred.

Five patients were non-responders. Two patients were primary non-responders and 3 patients had experienced LOR after achieving partial response initially. GLM treatment was discontinued after 6 months in 2 patients, after 9 months in another 2 patients, and after 18 months in the remaining patient. Attempts to procure response by reducing the treatment interval from 4 to 3 weeks preceded discontinuation of GLM. These attempts were unsuccessful.

### Visual acuity

BCVA did not change from baseline to final visit; this was true for the study eyes (*n* = 10), the affected fellow eyes (*n* = 7), and both groups taken together (*p* ≥ 0.05). Respective mean visual acuity values (logMAR) were 0.19 ± 0.28, 0.21 ± 0.30, and 0.20 ± 0.28, corresponding to a Snellen equivalent of around 0.63 each. Respective final visual acuity values were 0.27 ± 0.33, 0.19 ± 0.28, and 0.23 ± 0.31, corresponding to a Snellen equivalent of around 0.5 to 0.63.

### Corticosteroid-sparing potential

The mean dose of systemic corticosteroids was reduced from 0.19 mg/kg (range 0–0.52) at baseline to 0.09 mg/kg (range 0–0.27) at 1 month, to 0.08 mg/kg (range 0–0.23) at 3 months, and to 0.07 mg/kg (range 0–0.35) at 6 months. One patient received systemic steroids at 9 months at a dose of 0.9 mg/kg. No patient received systemic corticosteroids between assessments at 12 months and at 18 months. One patient received prednisolone 0.5 mg/kg when experiencing LOR at 18 months, as did another during a flare at 36 months.

With GLM treatment topical corticosteroid dose could be reduced from baseline (mean 5.3 drops/day) to at 1 month a mean of 4.3 drops/day, at 3 months a mean of 2.8 drops/day, at 6 months a mean of 4.7 drops/day, and at 12 months a mean of 2.3 drops/day. One patient received more than 2 drops of topical corticosteroids per day at 18 months and another at 36 months, but none beyond 42 months.

### Ocular complications

Ocular complications were present in 8 patients at the start of GLM treatment (see above). Macular edema resolved during GLM treatment in the 2 patients with this complication. New ocular complications were seen in 3 patients, including macular edema (*n* = 2) and synechiae (*n* = 2).

### Reason for ADA failure

ADA treatment was discontinued in 2 patients because of primary NR and because of LOR in 8. Of the 8, 5 initially manifested CR and 3 PR. ADA-drug monitoring, i.e. measurements of anti-ADA-antibody and drug-trough-level was performed in 7 patients and revealed immunogenicity as cause for LOR in 5 of them.

### Comparing response to GLM with response to ADA treatment

All patients who by 3 months responded to GLM had previously responded to ADA. Two patients showed a primary non-response to both drugs. These patients were subsequently switched to anti-interleukin-6 (IL-6) therapy, with a complete response to tocilizumab in both within 3 months.

**Fig. 1:**
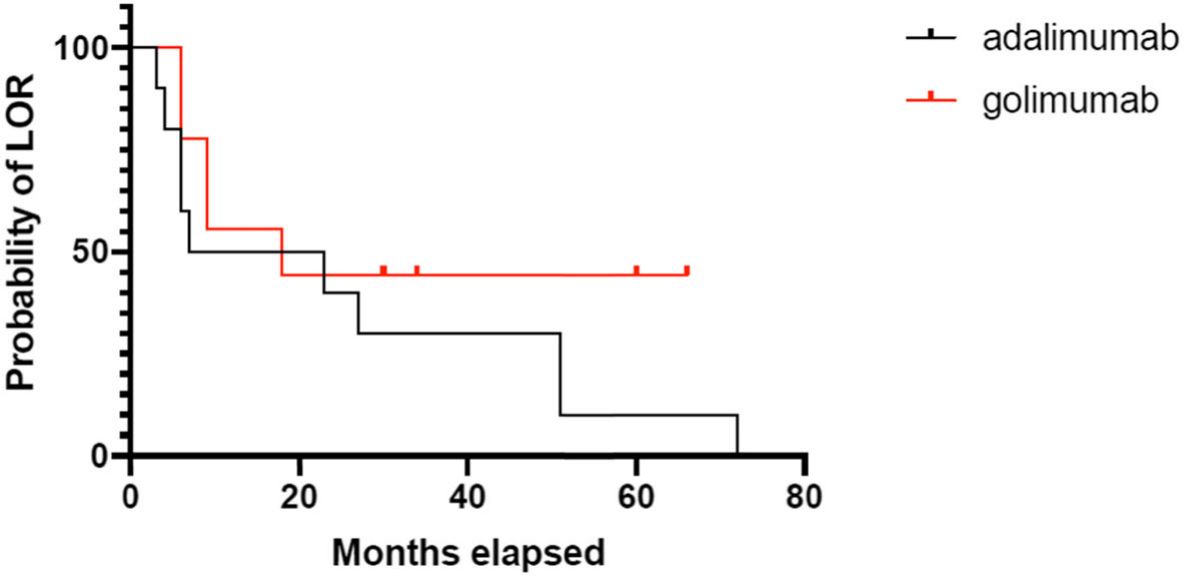
Time to treatment failure

Figure 1 shows time to treatment failure for GLM in relation to ADA in our study population. The median time to treatment failure for GLM was 26.8 months (95% CI 13.27 to 40.33).

### Side effects

GLM was well tolerated in 9 patients. In one patient viral infections were observed, including herpes genitalis and reactivation of cytomegalovirus. In this patient GLM was temporarily discontinued and restarted after 2 months.

### Arthritis

Four patients had active arthritis at the start of GLM treatment. In all 4 arthritis inactivity was initially achieved with GLM. One patient experienced relapse of both arthritis and uveitis at LOR.

## Discussion

ADA is the first-line biologic choice in refractory JIA-associated uveitis. However, patients in substantial numbers are discontinued ADA treatment for lack of efficacy [6, 7].

The options for managing failure of ADA treatment include switching to an alternative anti-TNFα-agent or switching to a drug with a different mode of action. Small case series have demonstrated that switching among anti-TNF agents, particularly from infliximab (IFX) to ADA, may be a reasonable option [10, 17]. Few data exist for the use of GLM in patients whose uveitis does not respond to ADA.

In our study, 8 of 10 patients in whom ADA failed responded initially to GLM. At their final visit GLM was effective in 5 patients. Four of these achieved sustained, long-term CR. This is in line with results of Palmou-Fontana et al., who reported that 4 out of 7 patients had achieved CR after being switched from various biological agents, including ADA, to GLM [12]. Misreocchi et al. reported uveitis inactivity in 14 of 17 patients who were switched from various biologic agents, including TNF- inhibitors, to GLM [13].

Neither of these studies investigated if the response to GLM was associated with the type of treatment failure using the prior anti-TNF agent; that is, neither study distinguished between primary NR and LOR.

We found that GLM was effective in our 8 patients in whom ADA was discontinued for LOR, whereas GLM was not effective in our 2 patients with primary non-response to ADA. A plethora of cytokines plays various roles in the pathogenesis of JIA-associated uveitis [18]. We infer that different patients require targeting of different cytokines for remission. This hypothesis is supported by the observation that both patients in whom GLM therapy failed rapidly achieved CR of uveitis after switching to anti-IL-6 therapy with tocilizumab.

An immune response to the agent employed is an important cause of LOR in the treatment of JIA-associated uveitis with anti-TNFα- antibodies. Immunogenicity of ADA underlay LOR in 35% of patients treated with ADA for JIA-associated uveitis [19], as reflected in SHARE initiative guidelines, which suggest drug-monitoring in case of loss of agent efficacy over time [5]. In this regard data exist for infliximab (IFX) and ADA [20]. Data for immunogenicity of GLM in JIA-associated uveitis are lacking. However, a systematic review of agent immunogenicity in different chronic inflammatory diseases found that anti-drug antibodies were detected in as many as 50% of patients in studies of ADA and IFX, but in < 20% in studies of GLM [21].

In our study, all 4 patients with a complete response to GLM had also initially achieved complete response to ADA. Drug monitoring was performed in 3 of these 4 patients and revealed neutralizing anti-ADA antibodies as cause for the LOR.

Concomitant immunosuppressive agents, such as methotrexate, azathioprine or leflunomide were shown to reduce the risk of antibody formation [19, 21]. Three of the 4 complete responders were intolerant of MTX and therefore received GLM as monotherapy. This is consistent with data that suggest lower immunogenicity of GLM than of ADA [21, 22].

As patients often come to dislike their concomitant immunosuppression, this may be an advantage of GLM that recommends it for preferential use and that can be extended.

Most of our 10 patients had severe recalcitrant uveitis, with multiple complications at the start of GLM treatment. Some of the patients had been treated for over a decade and were adults at time of start of GLM. Such patients tend to be less responsive to further treatment and prone to complications [23]. Macular edema was present in 2 patients; in both, it resolved under GLM treatment. We infer that patients with evident failure of ADA treatment should be switched to another agent early, before severe complications appear. Our data indicate that switching to GLM can also pay off in patients with long-lasting uveitis and severe complications.”

GLM treatment was generally well tolerated. We encountered no severe adverse events requiring discontinuation of the drug. GLM is approved for polyarticular JIA, with a long-term safety record like that of ADA.

Although our study is limited by the retrospective design and the relatively small number of patients, owing to the rarity of JIA, our data show that GLM is efficacious in long-term treatment of patients with JIA-associated uveitis in whom LOR to ADA has occurred.

## Conclusion

GLM is an effective treatment option in a subset of patients with JIA-associated uveitis. Switching to GLM is more effective in patients with LOR to ADA than in patients without a primary response to ADA. Primary non-responders might instead benefit from switching to a biologic agent with a different mode of action.

## Data Availability

All data generated or analyzed during this study are included in this published article.

## Abbreviations

ADA: Adalimumab
DMARD: Disease modifying antirheumatic drug
GLM: Golimumab
JIA: Juvenile idiopathic arthritis
MTX: Methotrexate
LOR: Loss of response
TNF: Tumor necrosis factor
